# Visceral adiposity index, lipid accumulation product and intracranial atherosclerotic stenosis in middle-aged and elderly Chinese

**DOI:** 10.1038/s41598-017-07811-7

**Published:** 2017-08-11

**Authors:** Rui Li, Qi Li, Min Cui, Zegang Ying, Lin Li, Tingting Zhong, Yingchao Huo, Peng Xie

**Affiliations:** 1 Department of Neurology, The First Affiliated Hospital of Chongqing Medical University, Chongqing, 400016 China; 2 Department of Neurology, Daping Hospital, Third Military Medical University, Chongqing, 400042 China; 3 South Ausralian Healthy and Medical Research Institute (SAHMRI), Adelaid, Australia

## Abstract

We investigated the association of the newly established lipid accumulation product (LAP) and visceral adiposity index (VAI) with intracranial atherosclerotic stenosis (ICAS) in middle-aged and elderly Chinese. From June 2012 to January 2013, consecutive patients ≥40 years of age who underwent cerebral vascular imaging for various medical reasons were enrolled in this study. Multivariate logistic regression models were used to evaluate the correlation of VAI and LAP with the risk of ICAS. In total, 845 patients were included in the study. The prevalence of ICAS gradually increased in females and in all patients with advancing tertiles of VAI or LAP. After adjusting for potential risks, both the VAI and LAP were related to ICAS in females [VAI: odds ratio (OR) = 3.25, 95% confidence interval (95%CI) = 1.17–9.03, P = 0.024; LAP: OR = 4.11, 95%CI = 1.39–12.12, P = 0.011; tertiles 3 vs. 1]. The sensitivity, specificity, and positive predictive value (PPV) were 74.7%, 45.5%, and 84.4% for VAI (cut-off: 1.71) and 79.3%, 40.5%, and 84.1% for LAP (cut-off: 23.99). The same relationships in males were not significant. Both VAI and LAP were closely associated with an increased risk of ICAS in middle-aged and elderly Chinese females.

## Introduction

Stroke is the second leading cause of mortality and morbidity in China and its prevalence is expected to increase further as the Chinese population ages. Intracranial atherosclerotic stenosis (ICAS) is an important cause of stroke^1^. Among more than 70% patients with ICAS, the 1-year recurrence rate of ischemic stroke (IS) or transient ischemic attack (TIA) is 23%^2^. ICAS is more common in China than in Western countries^3–5^. ICAS reportedly causes 33–40% of IS, and more than 50% of patients who develop TIA have ICAS as an associated risk factor^3, 4^. The reasons for the racial differences in the location of cerebral atherosclerosis are unclear, but they likely include differences in risk factors and the ethnicities of the study population^5^. ICAS has become a widespread public health issue and it imposes a significant social burden. Identifying patients with a high risk of ICAS may help to predict the risk of stroke.

The visceral adiposity index (VAI)^6^ and the lipid accumulation product (LAP)^7^ have been proposed as surrogate markers of visceral obesity. The LAP is a simple index based on the fasting triglyceride (TG) level and waist circumference (WC)^7^. The VAI is calculated from anthropometric parameters [WC and body mass index (BMI)] and metabolic indicators [high density lipoprotein cholesterol (HDL-C) and TG]^6^. Both the VAI and LAP are two reliable markers of central lipid accumulation, and their positive predictive values for cardiometabolic diseases has been demonstrated^6–13^. The VAI is significantly associated with insulin sensitivity, diabetes mellitus (DM)^8^, and visceral adipose tissue. Additionally, it is well documented that cardiovascular and cerebrovascular events are independently associated with the VAI^6^. The relationship between cardiovascular events^7, 9^ and the LAP has also been validated. For example, the LAP can be used to predict all-cause mortality in non-diabetic patients with cardiovascular risk factors^10^ and its accurate predictive value in insulin resistance (IR)^11^, DM^12^, and metabolic syndrome (MetS)^13^ has been reported.

Because of unhealthy sedentary lifestyles and ethnic variations, more visceral fat accumulation and less obesity are present among the Chinese than in Western populations^14^. As novel and easy-to-use indicators, the LAP and VAI have been shown to play a crucial role in assessing the risks of cardiovascular disease^6, 7^ and DM^8, 12^. However, the potential association of these indicators with the risk of ICAS has not been previously investigated. Therefore, in this study we evaluated whether the VAI and LAP can be used as indicators of the risk of ICAS in Chinese patients.

## Results

Of the 1055 consecutive patients who underwent cerebral vascular imaging and were initially included in our study, 210 were excluded because of missing anthropometric or laboratory data, acute stroke within 2 weeks, or non-atherosclerotic stenosis of an intracranial artery. Thus, 845 patients were finally enrolled. Six patients underwent magnetic resonance angiography (MRA), 18 patients were examined using digital subtraction angiography (DSA), and the remaining 821 patients by cerebral computed tomography angiography (CTA). Demographics of the patients are reported in Tables 1 and 2.

**Table 1:** Characteristics of patients stratified by VAI tertiles.

VAI Characteristics	Male			P value	Female			P value
	Tertile 1 (VAI < 1.07)	Tertile 2 (1.07 to 1.95)	Tertile 3 (>1.95)		Tertile 1 (VAI < 1.51)	Tertile 2 (1.51–2.56)	Tertile 3 (>2.56)	
No. of cases	130	133	132		149	150	151	
Age (years)	69.92 ± 9.30	67.20 ± 10.18	64.52 ± 9.47	P < 0.001	65.17 ± 9.18	64.74 ± 9.31	66.17 ± 9.15	0.384
Hypertension (%)	63 (48.5)	65 (48.9)	82 (62.1)	0.041	65 (43.6)	78 (52.0)	77 (51.0)	0.286
Diabetes Mellitus (%)	18 (13.8)	23 (17.3)	40 (30.3)	0.002	18 (12.1)	17 (11.3)	34 (22.5)	0.011
Coronary heart disease (%)	51 (39.2)	61 (45.9)	63 (47.7)	0.347	43 (28.9)	64 (42.7)	59 (39.1)	0.037
Previous stroke (%)	32 (24.6)	32 (24.1)	30 (22.7)	0.934	18 (12.1)	20 (13.3)	16 (10.6)	0.765
Current smoking (%)	39 (30.0)	51 (38.3)	56 (42.4)	0.105	1 (0.7)	2 (1.3)	3 (2.0)	0.875
Daily drinking, n (%)	19 (14.6)	32 (24.1)	28 (21.2)	0.146	3 (2.0)	2 (1.3)	1 (0.7)	0.460
Body mass index (kg/m^2^)	20.19 ± 2.98	23.47 ± 3.05	24.69 ± 2.74	P < 0.001	22.74 ± 3.37	23.55 ± 3.35	24.72 ± 3.40	P < 0.001
Waist circumference (cm)	79.75 ± 8.27	84.49 ± 8.27	88.38 ± 8.06	P < 0.001	78.65 ± 8.57	82.03 ± 9.15	86.46 ± 8.56	P < 0.001
Systolic blood pressure (mmHg)	134.85 ± 17.36	133.98 ± 17.39	133.15 ± 17.28	0.730	134.17 ± 16.47	133.31 ± 17.66	135.60 ± 16.38	0.490
Diastolic blood pressure (mmHg)	82.15 ± 9.63	81.94 ± 10.17	81.64 ± 10.48	0.921	80.97 ± 10.05	78.77 ± 10.39	79.46 ± 9.91	0.158
Fasting plasma glucose (mmol/L)	4.87 (4.48–5.47)	4.95 (4.49–5.49)	5.26 (4.70–6.57)	P < 0.001	4.98 (4.60–5.61)	4.99 (4.53–5.53)	5.21 (4.72–6.12)	0.003
Total cholesterol (mmol/L)	4.30 ± 0.93	4.45 ± 0.95	4.80 ± 1.11	P < 0.001	5.01 ± 1.00	5.06 ± 1.04	5.21 ± 1.04	0.192
Triglyceride (mmol/L)	0.73 (0.58–0.86)	1.15 (1.00–1.33)	2.07 (1.68–2.82)	P < 0.001	0.87 (0.72–1.01)	1.30 (1.14–1.51)	2.16 (1.74–2.63)	P < 0.001
HDL-C (mmol/L)	1.31 (1.12–1.46)	1.02 (0.93–1.17)	0.90 (0.80–1.04)	P < 0.001	1.52 (1.34–1.72)	1.24 (1.09–1.41)	1.06 (0.91–1.17)	P < 0.001
LDL-C (mmol/L)	2.20 ± 0.63	2.46 ± 0.65	2.61 ± 0.74	P < 0.001	2.60 ± 0.73	2.72 ± 0.71	2.88 ± 0.73	0.003
FRS	5.97 ± 2.52	6.95 ± 2.55	7.73 ± 2.87	P < 0.001	6.79 ± 3.21	8.30 ± 3.45	10.86 ± 3.47	P < 0.001

Continuous variables were analysed with one-way analysis of variance or the Kruskal-Wallis test, and categorical variables were analysed with the chi-square test or Fisher’s exact test. Results are expressed as mean ± SD for variables with a normal distribution, as median and interquartile range for variables with a skewed distribution, or as number with percentage for categorical variables. SD, standard deviation; HDL-C, high density lipoprotein cholesterol; LDL-C, low density lipoprotein cholesterol; VAI, visceral adiposity index; FRS, Framingham risk score.

**Table 2:** Characteristics of patients stratified by LAP tertiles.

LAP Characteristics	Male			P value	Female			P value
	Tertile 1 (LAP < 15.0)	Tertile 2 (15.0 to 31.39)	Tertile 3 (>31.39)		Tertile 1 (LAP < 22.17)	Tertile 2 (22.17–43.86)	Tertile 3 (>43.86)	
No. of cases	132	132	131		150	150	150	
Age (years)	69.36 ± 9.98	66.97 ± 9.75	65.26 ± 9.54	0.003	64.87 ± 9.27	65.23 ± 9.75	66.00 ± 8.61	0.558
Hypertension (%)	57 (43.2)	72 (54.5)	81 (61.8)	0.009	59 (39.3)	77 (51.3)	84 (56.0)	0.012
Diabetes Mellitus (%)	14 (10.6)	28 (21.2)	39 (29.8)	0.001	15 (10.0)	24 (16.0)	30 (20.0)	0.054
Coronary heart disease (%)	51 (38.6)	55 (41.7)	69 (52.7)	0.055	44 (29.3)	62 (41.3)	60 (40)	0.062
Previous stroke (%)	24 (18.2)	42 (31.8)	28 (21.4)	0.025	19 (12.7)	19 (12.7)	16 (10.7)	0.827
Current smoking (%)	45 (34.1)	46 (34.8)	55 (42.0)	0.343	1 (0.7)	1 (0.7)	4 (2.7)	0.378
Daily drinking, n (%)	26 (19.7)	18 (13.6)	35 (26.7)	0.030	3 (2.0)	2 (1.3)	1 (0.7)	0.875
Body mass index (kg/m^2^)	21.02 ± 2.50	23.79 ± 2.41	25.57 ± 2.49	P < 0.001	21.38 ± 2.63	23.79 ± 2.88	25.86 ± 3.28	P < 0.001
Waist circumference (cm)	75.76 ± 5.41	85.21 ± 5.61	91.79 ± 6.89	P < 0.001	74.51 ± 5.62	82.79 ± 6.81	89.88 ± 8.02	P < 0.001
Systolic blood pressure (mmHg)	133.22 ± 17.49	134.83 ± 17.28	133.92 ± 17.27	0.753	132.63 ± 15.91	135.23 ± 18.82	135.24 ± 15.54	0.302
Diastolic blood pressure (mmHg)	81.13 ± 9.83	82.59 ± 9.84	82.01 ± 10.59	0.496	79.02 ± 9.42	80.07 ± 10.99	80.10 ± 9.96	0.576
Fasting plasma glucose (mmol/L)	4.80 (4.43–5.35)	5.03 (4.54–5.43)	5.33 (4.71–7.02)	P < 0.001	4.92 (4.53–5.48)	5.12 (4.68–5.82)	5.11 (4.70–5.86)	0.005
Total cholesterol (mmol/L)	4.25 ± 0.97	4.34 ± 0.94	4.97 ± 0.99	P < 0.001	4.86 ± 1.00	5.16 ± 1.03	5.27 ± 1.02	0.001
Triglyceride (mmol/L)	0.76 (0.59–0.97)	1.10 (0.91–1.38)	2.07 (1.53–2.82)	P < 0.001	0.89 (0.72–1.09)	1.30 (1.06–1.62)	2.10 (1.62–2.64)	P < 0.001
HDL-C (mmol/L)	1.20 (1.04–1.44)	1.02 (0.90–1.21)	0.96 (0.84–1.11)	P < 0.001	1.37 (1.15–1.61)	1.29 (1.09–1.50)	1.11 (0.98–1.27)	P < 0.001
LDL-C (mmol/L)	2.21 ± 0.66	2.39 ± 0.68	2.67 ± 0.67	P < 0.001	2.57 ± 0.66	2.74 ± 0.77	2.88 ± 0.73	0.001
FRS	6.00 ± 2.60	6.93 ± 2.53	7.73 ± 2.83	P < 0.001	7.29 ± 3.59	8.39 ± 3.74	10.29 ± 3.36	P < 0.001

Continuous variables were analysed with one-way analysis of variance or the Kruskal-Wallis test, and categorical variables were analysed with the chi-square test or Fisher’s exact test. Results are expressed as mean ± SD for variables with a normal distribution, as median and interquartile range for variables with a skewed distribution, or as number with percentage for categorical variables. SD, standard deviation; HDL-C, high density lipoprotein cholesterol; LDL-C, low density lipoprotein cholesterol; LAP, lipid accumulation product; FRS, Framingham risk score.

### Characteristics of patients stratified by VAI tertiles

Patients of each sex were divided into tertiles based on the VAI (Table 1). In males, significant differences were found among VAI tertiles in age, hypertension, DM, BMI, WC, fasting plasma glucose (FPG), TG, total cholesterol (TC), low density lipoprotein cholesterol (LDL-C), HDL-C, and Framingham risk score (FRS) (P < 0.05). In females, significant differences were observed among VAI tertiles in DM, coronary heart disease (CHD), BMI, WC, FPG, FRS, HDL-C, LDL-C, and TG (P < 0.05). Males in the highest VAI tertile showed a higher prevalence of hypertension and DM, a higher BMI, WC, FPG, TG, TC, LDL-C, FRS, and lower age than males in the lowest VAI tertile. Females in the highest VAI tertile showed a higher prevalence of DM and CHD and a higher BMI, WC, FPG, FRS, TG, and LDL-C than those in the lowest VAI tertile. The HDL-C meaningfully decreased across increasing VAI tertiles in all patients (P < 0.001).

### Characteristics of patients stratified by LAP tertiles

Patients of each sex were divided into tertiles based on the LAP (Table 2). In males, significant differences were observed among the LAP tertiles in age, hypertension, DM, previous stroke, daily drinking, WC, BMI, FPG, FRS, TG, TC, LDL-C, and HDL-C (P < 0.05). In females, significant differences were observed among the LAP tertiles in hypertension, BMI, WC, FPG, FRS, TG, TC, LDL-C, and HDL-C (P < 0.05). Among all patients, LAP tertile 3 had a higher prevalence of hypertension and a higher BMI, WC, FPG, FRS, TC, TG, and LDL-C than LAP tertiles 2 and 1. HDL-C was negatively associated with the LAP in all patients (P < 0.001). Among males, a higher prevalence of DM and lower age were associated with higher LAP tertiles (P < 0.05).

### Prevalence of ICAS according to VAI and LAP in different genders

**Figure 1:**
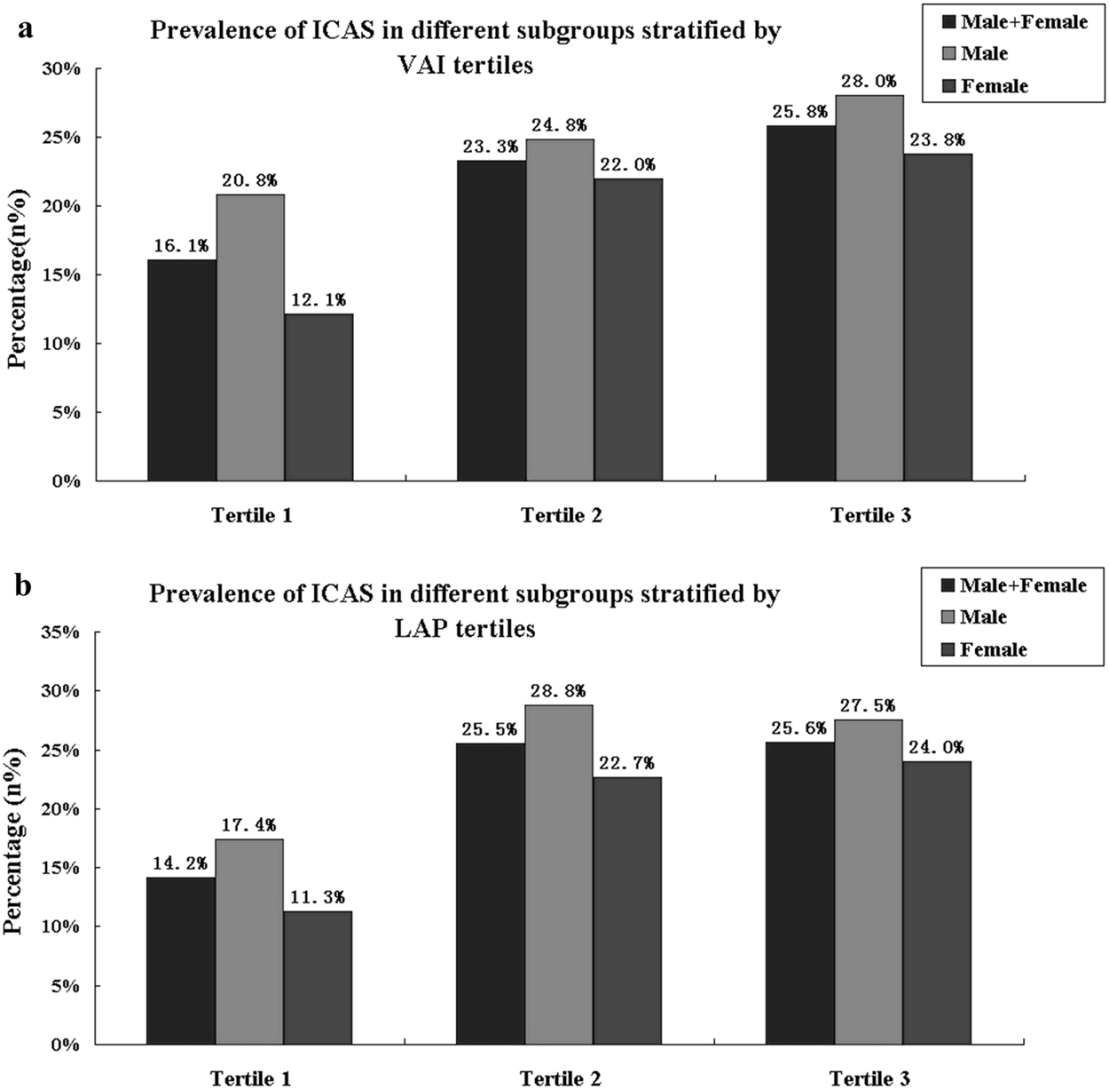
Prevalence of ICAS by VAI (**a**) and LAP (**b**) in males and females. The prevalence of ICAS was compared among the three tertiles of for the VAI (Male + Female, P = 0.016; Male, P = 0.392; Female, P = 0.022) and LAP (Male + Female, P = 0.001; Male, P = 0.064; Female, P = 0.009). ICAS, intracranial atherosclerotic stenosis; LAP, lipid accumulation product; VAI, visceral adiposity index.

Figure 1(a) shows that the prevalence of ICAS in VAI tertiles 1, 2 and 3 were 20.8%, 24.8% and 28.0% in males; 12.1%, 22.0% and 23.8% in females; 16.1%, 23.3% and 25.8% in all patients (males and females), respectively. As the VAI tertiles increased, a higher prevalence of ICAS was found within each sex. A significant difference in the prevalence of ICAS according to the VAI tertiles was observed in females (P = 0.022) and all patients (P = 0.016), but not in males (P = 0.392).

As illustrated in Fig. 1(b), the prevalence of ICAS gradually increased with higher LAP tertiles in females (11.3%, 22.7% and 24.0%; P = 0.009) and in all patients (14.2%, 25.5% and 25.6%; P = 0.001). However, this trend was not observed in males (17.4%, 28.8% and 27.5%; P = 0.064).

### VAI and LAP in different genders according to presence or absence of ICAS

**Figure 2:**
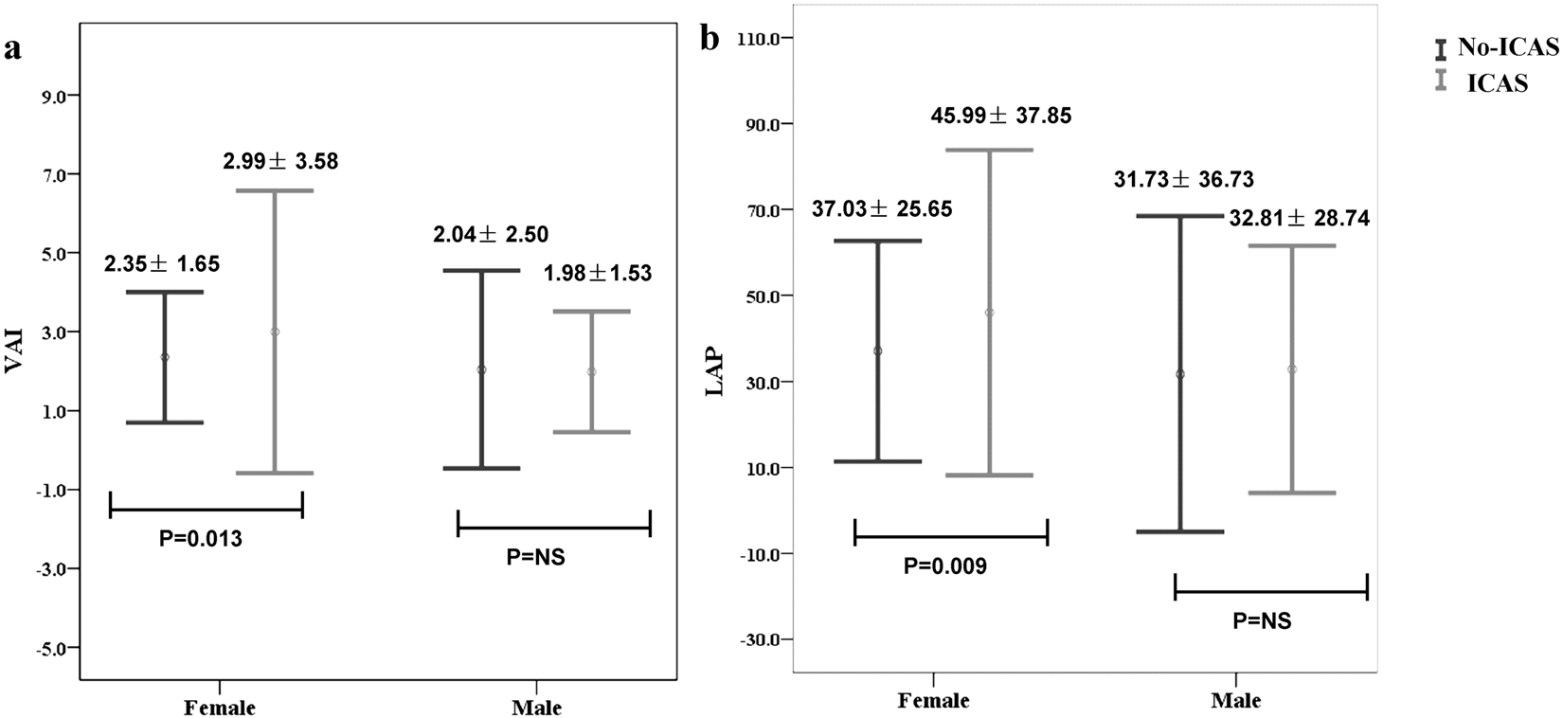
VAI (**a**) and LAP (**b**) in different genders according to presence or absence of ICAS. Data are mean ± SD. Error bars show SD. SD, standard deviation; LAP, lipid accumulation product; VAI, visceral adiposity index; ICAS, intracranial atherosclerotic stenosis; No-ICAS, no intracranial atherosclerotic stenosis.

As shown in Fig. 2, in females, those with ICAS (mean ± standard deviation) had higher VAI [2.99 ± 3.58 with ICAS, 2.35 ± 1.65 with no intracranial atherosclerotic stenosis (No-ICAS), P = 0.013] and LAP (45.99 ± 37.85 with ICAS, 37.03 ± 25.65 with No-ICAS, P = 0.009) than those with No-ICAS. However, significant differences were not found in males (VAI, P = 0.838; LAP, P = 0.792).

### Relationship between VAI and ICAS in different genders

The age-adjusted model demonstrated that the VAI correlated positively with ICAS in both sexes (Table 3). In females, compared with the lowest tertile (VAI tertile 1), the odds ratios (ORs) for ICAS in tertiles 2 and 3 were 2.29 [95% confidence interval (95%CI) = 1.18–4.42, P = 0.014] and 2.27 (95%CI = 1.19–4.35, P = 0.013), respectively. In males, the highest tertile (VAI tertile 3) was significantly associated with ICAS (OR = 1.94, 95%CI = 1.07–3.53, P = 0.03). After further adjustment for age, DM, hypertension, CHD, previous stroke, current smoking, daily drinking, BMI, HDL-C, FPG, WC, systolic blood pressure and diastolic blood pressure, an elevated risk of ICAS in association with the VAI was evident in females (VAI tertile 2: OR = 3.50, 95%CI = 1.48–8.31, P = 0.004; VAI tertile 3: OR = 3.25, 95%CI = 1.17–9.03, P = 0.024 vs. tertile 1). However, VAI tertile did not impact on the risk of ICAS in men (VAI tertile 2: OR = 1.25, 95%CI = 0.60–2.62, P = 0.547; VAI tertile 3: OR = 1.38, 95%CI = 0.58–3.26, P = 0.468 vs. tertile 1).

**Table 3:** Relationship between VAI and ICAS in different genders.

VAI	Model one^a^		Model two^b^	
	OR(95% CI)	P value	OR(95% CI)	P value
**Male**				
Tertile one	Reference		Reference	
Tertile two	1.43(0.79–2.59)	0.237	1.25(0.60–2.62)	0.547
Tertile three	1.94(1.07–3.53)	0.030	1.38(0.58–3.26)	0.468
**Female**				
Tertile one	Reference		Reference	
Tertile two	2.29(1.18–4.42)	0.014	3.50(1.48–8.31)	0.004
Tertile three	2.27(1.19–4.35)	0.013	3.25(1.17–9.03)	0.024

^a^Adjusted for age; ^b^Based on Model one, Model two was further adjusted for diabetes mellitus, hypertension, coronary heart disease, previous stroke, current smoking, daily drinking, body mass index, HDL-C, fasting plasma glucose, waist circumference, systolic blood pressure and diastolic blood pressure. HDL-C, high density lipoprotein cholesterol; VAI, visceral adiposity index; ICAS, intracranial atherosclerotic stenosis.

### Relationship between LAP and ICAS in different genders

Compared with patients in LAP tertile 1, the age-adjusted OR for ICAS in tertile 3 was 2.24 (95%CI = 1.21–4.15, P = 0.010) in males and 2.52 (95%CI = 1.30–4.87, P = 0.006) in females. This significant effect was also present for both males and females in LAP tertile 2 (males: OR = 2.21, 95%CI = 1.21–4.04, P = 0.010; females: OR = 2.38, 95%CI = 1.22–4.64, P = 0.011).

After further adjustment for relevant confounding factors including age, DM, hypertension, CHD, previous stroke, current smoking, daily drinking, BMI, HDL-C, FPG, WC, systolic blood pressure and diastolic blood pressure, LAP tertile 3 (OR = 4.11, 95%CI = 1.39–12.12, P = 0.011) and LAP tertile 2 (OR = 2.86, 95%CI = 1.19–6.87, P = 0.018) demonstrated increased ICAS risk in females, when compared to individuals within the LAP tertile 1. In contrast, we found no such association in males (LAP tertile 2: OR = 1.86, 95%CI = 0.84–4.12, P = 0.126; LAP tertile 3: OR = 1.91, 95%CI = 0.70–5.23, P = 0.207 vs. tertile 1) (Table 4).

**Table 4:** Relationship between LAP and ICAS in different genders.

LAP	Model one^a^		Model two^b^	
	OR(95% CI)	P value	OR(95% CI)	P value
**Male**				
Tertile one	Reference		Reference	
Tertile two	2.21(1.21–4.04)	0.010	1.86(0.84–4.12)	0.126
Tertile three	2.24(1.21–4.15)	0.010	1.91(0.70–5.23)	0.207
**Female**				
Tertile one	Reference		Reference	
Tertile two	2.38(1.22–4.64)	0.011	2.86(1.19–6.87)	0.018
Tertile three	2.52(1.30–4.87)	0.006	4.11(1.39–12.12)	0.011

^a^Adjusted for age; ^b^Based on Model one, Model two was further adjusted for diabetes mellitus, hypertension, coronary heart disease, previous stroke, current smoking, daily drinking, body mass index, HDL-C, fasting plasma glucose, waist circumference, systolic blood pressure and diastolic blood pressure. LAP, lipid accumulation product; HDL-C, high density lipoprotein cholesterol; ICAS, intracranial atherosclerotic stenosis.

We further investigated the 821 patients only with CTA scanning. The results were consistent with those of the 845 patients overall, as shown in Tables 3 and 4 (Tables S1 and S2).

### Receiver operating characteristic analysis of relevant variables in predicting ICAS

We determined the ability of the VAI, LAP, FRS, BMI, TG, and HDL-C to predict ICAS in males and females, by analysing the receiver operating characteristic (ROC) curves (Table 5). In females, the areas under the ROC curves (AUCs) for the VAI (0.588, 95%CI = 0.527–0.649) and LAP (0.590, 95%CI = 0.527–0.654) were higher than those for the BMI, TG, and HDL-C, but all were lower than the AUC for the FRS (0.588, 95%CI = 0.527–0.649). In males, only the FRS had a significant predictive value for ICAS (AUC = 0.670, 95%CI = 0.611–0.729). For the AUC value, we further compared VAI and LAP with the FRS in females. The differences between these two variables and the FRS were significant (FRS vs. VAI, P = 0.001; FRS vs. LAP, P = 0.006). The optimal cut-off values for the VAI and LAP levels in females were 1.71 [sensitivity = 74.7%, specificity = 45.5%, positive predictive value (PPV) = 84.4%] and 23.99 (sensitivity = 79.3%, specificity = 40.5%, PPV = 84.1%) for LAP, respectively. The FRS (cut-off value: 9.5) had a higher specificity (67.0%) and PPV (88%), and a lower sensitivity (63.2%) than the respective VAI and LAP values.

**Table 5:** ROC analysis of relevant variables in predicting ICAS.

	Female		Male	
	AUC(95% CI)	P value	AUC(95% CI)	P value
VAI	0.588(0.527–0.649)	0.010	0.554(0.491–0.618)	0.108
LAP	0.590(0.527–0.654)	0.009	0.543(0.478–0.608)	0.204
FRS	0.698(0.639–0.756)	<0.001	0.670(0.611–0.729)	<0.001
Body mass index	0.515 (0.447–0.583)	0.667	0.533(0.468–0.598)	0.334
Triglyceride	0.555(0.492–0.618)	0.110	0.523(0.458–0.587)	0.499
HDL-C	0.422(0.359–0.485)	0.024	0.431(0.366–0.497)	0.042

ROC, receiver operating characteristic; AUC, area under the ROC curve; 95% CI: 95% confidence intervals; VAI, visceral adiposity index; LAP, lipid accumulation product; FRS, Framingham risk score; HDL-C, high density lipoprotein cholesterol; ICAS, intracranial atherosclerotic stenosis.

## Discussion

The present study assessed the association of two parameters of visceral obesity, VAI and LAP, with the occurrence of ICAS in the Chinese population. The results showed a gradual increase in ICAS with increasing VAI and LAP values in females. The ROC analysis confirmed the predictive value of the LAP and VAI in discriminating ICAS. After adjustment for age and potential risk factors, both the VAI and LAP were independently associated with ICAS in females but not consistently in males. The key finding of this study is that the LAP and VAI might be useful biomarkers for identifying females with ICAS.

China currently has an ageing population, which is associated with an increasing prevalence of cerebrovascular diseases. Additionally, racial distribution, socio-economic status, lifestyles, metabolic disorders and abdominal obesity differ between China and Western countries^14^. Considering the unhealthy diet and sedentary lifestyle in China^15^, abdominal obesity has become a characteristic of much of the population. Thus, investigation of the underlying surrogate markers of abdominal obesity, metabolic disorders and stroke risk in the Chinese population is of clinical importance^16^.

In recent years, the VAI and LAP have been investigated in patients with many cardiometabolic diseases^6, 7, 10, 12, 13, 17^. As surrogate markers, both the VAI and LAP can accurately differentiate between visceral adiposity and subcutaneous adiposity^18^. More particularly, visceral adiposity can be evaluated using the VAI instead of the traditional gold standard techniques of magnetic resonance imaging (MRI) and computed tomography (CT)^19^.

The VAI was initially introduced by Amato *et al*.^6^. TG, HDL-C, BMI and WC are central parameters of the VAI and are used to assess the adipose tissue distribution and physiological function. In a study of 1498 Caucasian patients undergoing primary care, Amato *et al*.^6, 20^ reported that the VAI has a close connection with cardiometabolic risk, elevated blood pressure, FPG, TG and low HDL-C. Additionally, both cardiovascular events and cerebrovascular events were shown to correlate positively with the VAI. Mohammadreza *et al*.^21^ proposed that the VAI as a reliable indicator in identifying the risk of cardiovascular disease in females. There is accumulating evidence of a remarkable interaction between the VAI and the risk of both DM^8^ and IR^19^ in the Asian populations. For instance, a study of 414 Saudi individuals demonstrated that the VAI could be used to evaluate the risk of impaired glucose metabolism^22^. Likewise, a remarkable link between hypertension and the VAI has been identified in the Chinese population^23^.

The LAP, initially described by Kahn^7^, is a novel index of visceral adiposity that is based on WC and the fasting TG level. Previous studies indicated that the LAP reflects certain vascular risk factors, includeing DM^12^, IR^24^, and hypertension^25^. Wakabayashi and Daimon^12^ identified a strong association between the LAP and DM in 10,170 Japanese subjects, and a study of 2,524 Chinese subjects demonstrated the stronger contribution to the IR index to the LAP than either BMI or WC. Moreover, data from 381 Caucasians indicated that the LAP is a precise index for predicting the outcome of the oral glucose tolerance test^26^. With regard to hypertension, one study of 36,536 Japanese men indicated a positive association between the LAP and blood pressure^25^. Notably, the LAP can also be used to predict cardiovascular diseases^27^, the incidence of cardiovascular events^28^, and all-cause mortality^9, 10^ in different ethnic populations. In addition, Ioachimescu *et al*.^10^ found that the LAP was associated with the mortality of nondiabetic patients with cardiovascular risks.

The connection between visceral obesity and atherosclerosis has also been extensively investigated in Asian and European populations. Yang *et al*.^29^ identified an important correlation between the VAI and increased arterial stiffness in Asian populations, while Zhang *et al*.^30^ found that the VAI was independently associated with CHD in the Chinese. The validity of the LAP was similarly confirmed in a Caucasian cohort^7^. Thus far, only one study has focused on the relationship between the VAI and atherosclerotic risk^31^. The authors analysed the association between the VAI and coronary artery calcium score in Korean adults and found that those with a high VAI had an increased risk of subclinical atherosclerosis. However, no studies have examined the association between the VAI and ICAS. The intracranial arteries contain higher antioxidant levels than the extracranial arteries and may be more vulnerable to risk factors leading to antioxidant depletion^32^. With a reduction in antioxidant protection, the intracranial arteries responded with accelerated atherogenesis to a greater degree than do the extracranial arteries. The progression can be explained by the reduced intracellular defences against oxygen radical –mediated processes^32^. Also, systemic factors (vascular risk factors or genetic factors) and local factors (haemodynamic or structural factors) may differ between the intracranial and extracranial arteries^5^.

The present study revealed a sex-specific association between visceral adiposity and ICAS. One possible explanation may be that the main indices of visceral adiposity have sex-related differences. A previous study demonstrated that visceral adiposity was more strongly correlated with DM in females than males^12^. Therefore, the DM-associated risk of ICAS might also show a stronger trend in females.

Hormonal factors may represent another underlying pathophysiologic link between visceral adiposity and ICAS in females. Hormone replacement therapy has been shown to decrease both the formation of atherosclerotic plaques in animal models and the incidence of cardiovascular disease in females^33^. Most women in the present study were postmenopausal, with the physical and hormonal changes associated with menopause potentially contributing to the link between visceral adiposity and ICAS. The exact mechanism of these sex-related differences remains unclear, and warrant further investigation.

Most patients in the present study underwent evaluation by CTA. CTA provides high-quality images of the intracranial blood vessels using a non-invasive approach^34^. Compared with MRA, CTA examination is less susceptible to motion artefacts and haemodynamic effects and is used as a routine clinical examination for imaging of intracranial vessels^35^. CTA can reveal >50% ICAS with a high specificity (97.1%) and sensitivity (99.5%) comparable with DSA^36^. Thus, CTA has been selected as a credible, minimally invasive screening approach for intracranial arterial disease and occlusion^37^. MRA has a high sensitivity (92%) and specificity (91%) for the identification of ≥50% stenosis^38^. Because of the small sample size and similar accuracies of MRA and DSA for assessing ICAS, MRA and DSA were unlikely to have affected the reliability of our results.

Some risk factors and biomarker profiles (MetS, adiponectin and other classic risk factors) have been associated with ICAS^39–41^. However, the utility of LAP and VAI has not been investigated in previous studies. Based on the findings of the present study, LAP and VAI could be selected as new and simple predictors of ICAS. The VAI and LAP are more commonly used than adiponectin in clinical tests, and their determination requires fewer laboratory data than MetS. Use of the VAI and LAP can therefore decrease the amount of time required to evaluate patients for ICAS and alleviate the need for complex diagnostic testing. Compared with classic risk factors, the VAI and LAP are more informative regarding cardiometabolic risk and can be more quickly assessed. Additionally, these simple tools can provide patients with greater incentive for lifestyle changes because the effects of lifestyle changes on vascular risk can be easily tracked. However, it remains to be determined whether control of metabolic risk and visceral adiposity could help to reduce risk of ICAS.

For women, the FRS had the highest predictive value for ICAS. Using ROC analysis, we obtained the optimal cut-off values of the variables that identify ICAS (VAI: 1.71, LAP: 23.99, FRS: 9.5). Of these, VAI and LAP had good sensitivity and PPV but weak specificity. By comparison, the FRS had a higher specificity and PPV and a lower sensitivity. In clinical practice, in patients in whom the values are above the respective cut-offs, scanning for ICAS should be conducted. Moreover, the use of these cut-off values could enable the prediction of the increased risk of both ICAS and stroke. In their patients with a high VAI and high LAP, clinicians should be alert to the increased risk of both diseases. As simple, inexpensive tools allowing routine clinical measurement, the VAI and LAP can be used in the control and early intervention in patients with vascular risks.

Some limitations of our research should be mentioned and discussed. First, this was a single-centre, cross-sectional study. The number of patients receiving MRA or DSA scan was a relatively small sample size. In addition, we included both healthy subjects and patients with various diseases and disorders. The single-centre and single-ethnicity sample also prevents drawing conclusions regarding causality. Thus, prospective trials involving larger cohorts are required to confirm the accuracy of these findings in Asians and other ethnic groups. Second, our study included patients with previous IS or TIA. Patient selection was hospital-based, and the prevalence of intracranial atherosclerosis was high. Moreover, inpatients appeared to have more atherosclerotic comorbidities, DM and hypertension than community-based populations. Therefore, research based on healthy populations should be performed in the future. Third, ICAS was defined as the presence of ≥50% stenosis in our study. Patients with ICAS of <50% were excluded from the ICAS group, which may have led to potential selection bias. Fourth, extracranial atherosclerosis was not evaluated in our study. Extracranial atherosclerosis should be considered when evaluating the risk of cerebral large arterial atherosclerosis. Finally, several potential risk factors such as diet, nutrition, endothelial dysfunction, menopausal status as well as socioeconomic factors were not collected and analysed in this study.

In conclusion, the present study has shown that two surrogate indicators of visceral adiposity, the VAI and LAP, may be associated with the risk of ICAS in females but not males among patients aged ≥40 years.

## Methods

### Study population

From June 2012 to January 2013, consecutive patients in the Neurology Department of Daping Hospital were included in this retrospective cross-sectional study. Relevant vascular neuroimaging examinations were performed in all patients for different medical reasons (dizziness, vertigo, headache, anxiety, sensorimotor disorder, routine health examinations, or complaints of neurological symptoms). Vascular neuroimaging included MRA, DSA and CTA. Eligible patients enrolled in this study were aged ≥40 years and long-term residents of local districts. The exclusion criteria were (1) a lack of ability or refusal to undergo vascular imaging, (2) missing clinical information or demographic or laboratory data, (3) non-atherosclerotic stenosis of an intracranial artery (arterial dissection, vasculitis, moyamoya disease, fibromuscular dysplasia or radiation-induced vasculopathy), and (4) onset of symptoms of acute stroke within 2 weeks. This study was approved by the Ethics Committee of Daping Hospital. All patients provided written informed consent. This study protocol was performed in accordance with the Declaration of Helsinki.

### Clinical data

We utilised a standardised interview with a specified questionnaire that included demographic data, medical history (hypertension, DM and cardiovascular disease), current smoking, drinking status and the drug use history. Information was obtained from the patients’ self-reports and credible records. Blood pressure was measured with an aneroid sphygmomanometer after resting for 10 minutes. Fasting blood samples were drawn from patients and sent to the central laboratory of Daping Hospital to measure the concentrations of TC, FPG, TG, LDL-C and HDL-C by standard enzymatic techniques using automatic biochemical analysers with commercially available reagents (DxC 800 chemistry analyser; Beckman Coulter Inc., Brea, CA, US). Quality control and standardisation were assured by professional staff at the central laboratory. BMI was calculated as weight divided by the square of height (kg/m^2^). WC was measured at the umbilical level after normal expiration. Current smoking was defined as consumption of ≥1 cigarette each day. Daily drinking was defined as alcohol consumption of ≥8 g each week. Physicians’ diagnostic reports regarding CHD or previous stroke were collected for each patient. The FRS was calculated based on age, sex, LDL-C, HDL-C, blood pressure, smoking status and history of DM^42^.

### Classification of ICAS

ICAS was defined as significant stenosis in anterior, middle or posterior cerebral artery, basilar artery, intracranial segments of internal carotid artery or vertebral artery. The degree of stenosis was assessed with the standardised method for ICAS^43^. The absence of stenosis was defined as ≥50% stenosis in the large intracranial vessels. No-ICAS was defined as <50% or no stenosis in intracranial arteries. Results were interpreted by two trained raters, and differences were resolved by joint discussion.

### Definition of VAI and LAP

The LAP was calculated in both sexes using following equations: [WC (cm) − 65] × TG (mmol/L) for males and [WC (cm) − 58] × TG (mmol/L) for females^7^. To avoid negative values, WC of <65 cm in males and <58 cm in females were redefined as 66 and 59 cm, respectively^44^.

The VAI was determined by sex-specific equations^5^: [WC/(39.68 + (1.88 × BMI))] × (TG/1.03) × (1.31/HDL) for males and [WC/(36.58 + (1.89 × BMI))] × (TG/0.81) × (1.52/HDL) for females. WC was calculated in cm and HDL-C and TG in mmol/L.

### Statistical analysis

Statistical analyses were performed by SPSS 18.0. Males and females were classified into three groups according to sex-specific tertiles of LAP or VAI. Quantitative data were compared with one-way analysis of variance or Student’s t test for normal distributions and with the Kruskal–Wallis test for skewed distributions. For normally distributed data, the results are presented as mean ± standard deviation. For data with a skewed distribution, the results are presented as median and interquartile range. Categorical data are expressed as percentages and were compared with Fisher’s exact test or Pearson’s chi square test.

The relationships between the tertiles of LAP or VAI and the risk of ICAS were evaluated with the chi square test. The diagnostic ability of ICAS was defined by the area under the curve in the ROC analyses. MedCalc version 11.4.2.0 (MedCalc Software bvba) was used to compare AUC values. The optimal cut-off value was determined by maximal Youden index. Logistic regression models were performed to evaluate this connection of the LAP and VAI with ICAS separately in males and females. Patients in the lowest tertile represented the reference category. Model one was adjusted for age, and Model two was adjusted for potential confounders. The results are shown as OR and 95%CI. A two-tailed P value of <0.05 was regarded as statistically significant.

## Electronic supplementary material

Supplementary Information

## References

[1] Qureshi AI, Caplan LR (2014). Intracranial atherosclerosis. Lancet.

[2] Chimowitz MI (2011). Stenting versus aggressive medical therapy for intracranial arterial stenosis. N Engl J Med.

[3] Huang HW (2007). Prevalence and risk factors of middle cerebral artery stenosis in asymptomatic residents in Rongqi County, Guangdong. Cerebrovasc Dis.

[4] Wong KS (1998). Intracranial stenosis in Chinese patients with acute stroke. Neurology.

[5] Kim JS, Bonovich D (2014). Research on Intracranial Atherosclerosis from the East and West: Why Are the Results Different?. J Stroke.

[6] Amato MC (2010). Visceral Adiposity Index: a reliable indicator of visceral fat function associated with cardiometabolic risk. Diabetes care.

[7] Kahn HS (2005). The “lipid accumulation product” performs better than the body mass index for recognizing cardiovascular risk: a population-based comparison. BMC Cardiovasc Disord.

[8] Du T, Sun X, Huo R, Yu X (2014). Visceral adiposity index, hypertriglyceridemic waist and risk of diabetes: the China Health and Nutrition Survey 2009. Int J Obes (Lond).

[9] Wehr E, Pilz S, Boehm BO, März W, Obermayer-Pietsch B (2011). The lipid accumulation product is associated with increased mortality in normal weight postmenopausal women. Obesity (Silver Spring).

[10] Ioachimescu AG, Brennan DM, Hoar BM, Hoogwerf BJ (2010). The lipid accumulation product and all-cause mortality in patients at high cardiovascular risk: a PreCIS database study. Obesity (Silver Spring, Md).

[11] Mirmiran P, Bahadoran Z, Azizi F (2014). Lipid accumulation product is associated with insulin resistance, lipid peroxidation, and systemic inflammation in type 2 diabetic patients. Endocrinol Metab (Seoul).

[12] Wakabayashi I, Daimon T (2014). A strong association between lipid accumulation product and diabetes mellitus in japanese women and men. J Atheroscler Thromb.

[13] Xiang S (2013). Lipid accumulation product is related to metabolic syndrome in women with polycystic ovary syndrome. Exp Clin Endocrinol Diabetes.

[14] Nazare JA (2012). Ethnic influences on the relations between abdominal subcutaneous and visceral adiposity, liver fat, and cardiometabolic risk profile: the International Study of Prediction of Intra-Abdominal Adiposity and Its Relationship With Cardiometabolic Risk/Intra-Abdominal Adiposity. Am J Clin Nutr.

[15] Du H (2013). Physical activity and sedentary leisure time and their associations with BMI, waist circumference, and percentage body fat in 0.5 million adults: the China Kadoorie Biobank study. Am J Clin Nutr.

[16] Cheng P (2015). BMI Affects the Relationship between Long Chain N-3 Polyunsaturated Fatty Acid Intake and Stroke Risk: a Meta-Analysis. Scientific reports.

[17] Wanderley Rocha, D. R., Jorge. A. R., Braulio, V. B., Arbex, A. K. & Marcadenti, A. Visceral adiposity measurements, metabolic and inflammatory profile in obese patients with and without type 2 diabetes mellitus: a cross-sectional analysis. *Current diabetes reviews* (2015). 10.2174/1573399812666151015115924 26467189

[18] Roriz AK (2014). Evaluation of the accuracy of anthropometric clinical indicators of visceral fat in adults and elderly. PLoS One.

[19] Oh JY, Sung YA, Lee HJ (2013). The visceral adiposity index as a predictor of insulin resistance in young women with polycystic ovary syndrome. Obesity.

[20] Amato MC, Giordano C, Pitrone M, Galluzzo A (2011). Cut-off points of the visceral adiposity index (VAI) identifying a visceral adipose dysfunction associated with cardiometabolic risk in a Caucasian Sicilian population. Lipids Health Dis.

[21] Mohammadreza B, Farzad H, Davoud K (2012). Fereidoun Prof AF. Prognostic significance of the complex “Visceral Adiposity Index” vs. simple anthropometric measures: Tehran lipid and glucose study. Cardiovasc Diabetol.

[22] Al-Daghri NM (2013). Visceral adiposity index is highly associated with adiponectin values and glycaemic disturbances. Eur J Clin Invest.

[23] Ding Y (2015). Significantly increased visceral adiposity index in prehypertension. PLoS One.

[24] Xia C (2012). Lipid accumulation product is a powerful index for recognizing insulin resistance in non-diabetic individuals. Eur J Clin Nutr.

[25] Wakabayashi I (2015). Associations of blood lipid-related indices with blood pressure and pulse pressure in middle-aged men. Metab Syndr Relat Disord.

[26] Malavazos AE (2015). The “lipid accumulation product” is associated with 2-hour postload glucose outcomes in overweight/obese subjects with nondiabetic fasting glucose. Int J Endocrinol.

[27] Nascimento JX (2015). Importance of lipid accumulation product index as a marker of CVD risk in PCOS women. Lipids Health Dis.

[28] Hosseinpanah F (2016). Lipid accumulation product and incident cardiovascular events in a normal weight population: Tehran Lipid and Glucose Study. Eur J Prev Cardiol.

[29] Yang F (2014). Visceral adiposity index may be a surrogate marker for the assessment of the effects of obesity on arterial stiffness. PLoS One.

[30] Zhang X (2013). Visceral adiposity and risk of coronary heart disease in relatively lean Chinese adults. Int J Cardiol.

[31] Park HJ (2016). Increased risk of subclinical atherosclerosis associated with high visceral adiposity index in apparently healthy Korean adults: the Kangbuk Samsung Health Study. Ann Med.

[32] D’Armiento FP (2001). Age-related effects on atherogenesis and scavenger enzymes of intracranial and extracranial arteries in men without classic risk factors for atherosclerosis. Stroke.

[33] Ouyang P, Michos ED, Karas RH (2006). Hormone replacement therapy and the cardiovascular system lessons learned and unanswered questions. J Am Coll Cardiol.

[34] Bash S (2005). Intracranial vascular stenosis and occlusive disease: evaluation with CT angiography, MR angiography, and digital subtraction angiography. Am J Neuroradiol.

[35] Li Q, Lv F, Yao G, Li Y, Xie P (2014). 64-section multidetector CT angiography for evaluation of intracranial aneurysms: comparison with 3D rotational angiography. Acta Radiol.

[36] Nguyen-Huynh MN (2008). How accurate is CT angiography in evaluating intracranial atherosclerotic disease?. Stroke.

[37] Li Q, Lv F, Wei Y, Luo T, Xie P (2013). Automated subtraction CT angiography for visualization of the whole brain vasculature: a feasibility study. Acad Radiol.

[38] Hirai T (2002). Prospective evaluation of suspected stenoocclusive disease of the intracranial artery: combined MR angiography and CT angiography compared with digital subtraction angiography. Am J Neuroradiol.

[39] Bang OY (2005). Association of the metabolic syndrome with intracranial atherosclerotic stroke. Neurology.

[40] Bang OY (2007). Adiponectin levels in patients with intracranial atherosclerosis. Neurology.

[41] Lopez-Cancio E (2012). Biological signatures of asymptomatic extra-and intracranial atherosclerosis: the Barcelona-Asia (Asymptomatic Intracranial Atherosclerosis) study. Stroke.

[42] Wilson PW (1998). Prediction of coronary heart disease using risk factor categories. Circulation.

[43] Samuels OB, Joseph GJ, Lynn MJ, Smith HA, Chimowitz MI (2000). A standardized method for measuring intracranial arterial stenosis. Am J Neuroradiol.

[44] Du T, Yu X, Zhang J, Sun X (2015). Lipid accumulation product and visceral adiposity index are effective markers for identifying the metabolically obese normal-weight phenotype. Acta Diabetol.

