# Supplementary material for: Visceral adiposity index, lipid accumulation product and intracranial atherosclerotic stenosis in middle-aged and elderly Chinese
Source: Sci Rep. 2017 Aug 11;7:7951. doi: 10.1038/s41598-017-07811-7 (PMC5554161; doi:10.1038/s41598-017-07811-7)
Supplement: Supplementary file 1 — Supplementary Information [file 41598_2017_7811_MOESM1_ESM.doc]

**Visceral adiposity index, lipid accumulation product and intracranial atherosclerotic stenosis** **in middle-aged and elderly Chinese**

**(Supplementary Materials)**

Rui Li^1^, Qi Li^1^, Min Cui^2^, Zegang Ying^2^, Lin Li^2^, Tingting Zhong^2^, Yingchao Huo^2^, Peng Xie^1,3^ *****

^1^Department of Neurology, The First Affiliated Hospital of Chongqing Medical University, Chongqing 400016, China

^2^Department of Neurology, Daping Hospital, Third Military Medical University, Chongqing 400042, China

^3^South Ausralian Healthy and Medical Research Institute (SAHMRI), Adelaid, Australia

***Corresponding author and reprints:**

**Peng Xie, Department of Neurology, The First Affiliated Hospital of Chongqing Medical University, No.1 Youyi Road, Chongqing 400042, China. Tel: +86-23-68485490; Fax: +86-23-68485111; E-mail:** [xiepeng@cqmu.edu](mailto:xiepeng@cqmu.edu)**.cn**

**Supplementary Materials**

**Tables (Supplementary, Total 2)**

**Table S1.** Relationship between VAI and ICAS in patients underwent cerebral CTA

| VAIV VAI | Model one ^a^ | |  | Model two ^b^ | |
| --- | --- | --- | --- | --- | --- |
|  | OR(95% CI) | P value |  | OR(95% CI) | P value |
| Male |  |  |  |  |  |
| Tertile one | Reference |  |  | Reference |  |
| Tertile two | 1.38(0.76-2.51) | 0.287 |  | 1.20(0.57-2.53) | 0.626 |
| Tertile three | 1.97(1.08-3.58) | 0.027 |  | 1.36(0.57-3.23) | 0.486 |
|  |  |  |  |  |  |
| Female |  |  |  |  |  |
| Tertile one | Reference |  |  | Reference |  |
| Tertile two | 2.54(1.30-4.98) | 0.006 |  | 3.84(1.60-9.24) | 0.003 |
| Tertile three | 2.50(1.29-4.85) | 0.007 |  | 3.56(1.26-10.00) | 0.016 |

^a^Adjusted for age; ^b^Based on Model one, Model two was further adjusted for diabetes mellitus, hypertension, coronary heart disease, previous stroke, current smoking, daily drinking, body mass index, HDL-C, fasting plasma glucose, waist circumference, systolic blood pressure and diastolic blood pressure. HDL-C, high density lipoprotein cholesterol; CTA, computed tomography angiography; VAI, visceral adiposity index; ICAS, intracranial atherosclerotic stenosis

**Table S2.** Relationship between LAP and ICAS in patients underwent cerebral CTA

| VAIL LAP | Model one ^a^ | |  | Model two ^b^ | |
| --- | --- | --- | --- | --- | --- |
|  | OR(95% CI) | P value |  | OR(95% CI) | P value |
| Male |  |  |  |  |  |
| Tertile one | Reference |  |  | Reference |  |
| Tertile two | 2.13(1.16-3.91) | 0.015 |  | 1.80(0.81-4.00) | 0.151 |
| Tertile three | 2.21(1.19-4.09) | 0.012 |  | 1.78(0.65-4.84) | 0.261 |
|  |  |  |  |  |  |
| Female |  |  |  |  |  |
| Tertile one | Reference |  |  | Reference |  |
| Tertile two | 2.63(1.33-5.19) | 0.005 |  | 3.01(1.25-7.26) | 0.014 |
| Tertile three | 2.76(1.41-5.41) | 0.003 |  | 4.24(1.44-12.47) | 0.009 |

^a^Adjusted for age; ^b^Based on Model one, Model two was further adjusted for diabetes mellitus, hypertension, coronary heart disease, previous stroke, current smoking, daily drinking, body mass index, HDL-C, fasting plasma glucose, waist circumference, systolic blood pressure and diastolic blood pressure. CTA, computed tomography angiography; LAP, lipid accumulation product; HDL-C, high density lipoprotein cholesterol; ICAS, intracranial atherosclerotic stenosis
